# Technology Assessment vs. Technology Appraisal—How to Strengthen the Science/Value Dichotomy with EU HTA?

**DOI:** 10.3390/jmahp12040028

**Published:** 2024-11-18

**Authors:** Sandro Gsteiger, Heiner C. Bucher, James Ryan, Jörg Ruof

**Affiliations:** 1 F. Hoffmann-La Roche AG, 4070 Basel, Switzerland; 2 Division of Clinical Epidemiology, University Hospital Basel and University of Basel, 4051 Basel, Switzerland; 3 Astra Zeneca, Cambridge CB2 8PA, UK; 4 Secretariat of the European Access Academy, 4059 Basel, Switzerland; 5 Institute for Epidemiology, Social Medicine and Health System Research, Medical School of Hanover, 30625 Hanover, Germany

**Keywords:** EU HTA regulation, EU HTA process, innovative health technologies, patient access, European access environment, HTA procedures, EU HTA initiative, HTA bodies, European access academy, market access society, HTA community

## Abstract

Many countries around the world use health technology assessment (HTA) to inform reimbursement and pricing decisions. HTA is often split into two steps, called assessment and appraisal. While the term HTA itself has been defined by international consortia, there is heterogeneity in the way different stakeholders use the terms assessment and appraisal. This creates ambiguity regarding which activities are included in technology assessment. With the new EU HTA Regulation, the HTA community should urgently seek to clarify the distinction between assessment and appraisal, as the regulation aims to centralize the clinical part of technology assessment at the European level. Failure to clarify this terminology will put the ambition of the regulation such as increased efficiency and reduction in duplication at risk. In this article, we argue that the distinction between assessment and appraisal should be seen as a science/value dichotomy. We discuss the transition from centralized assessment activities to country-level appraisal, which should culminate in a categorization of the overall added benefit in a local context. Finally, we touch on the important dimension of uncertainty always present in medical decision making.

## 1. Introduction

Many countries around the world use health technology assessment (HTA) to inform pricing and reimbursement decisions [1,2,3]. Typically, countries follow a structured HTA process involving two main stages: a first scientific evaluation, followed by a formal valuation step. These stages are often referred to as technology assessment and technology appraisal, respectively [4]. The term HTA itself has been defined by a task force led by the International Network of Agencies for Health Technology Assessment (INAHTA) and Health Technology Assessment International (HTAi) as “a multidisciplinary process that uses explicit methods to determine the value of a health technology at different points in its lifecycle. The purpose is to inform decision-making in order to promote an equitable, efficient, and high-quality health system” [5]. However, there is considerable heterogeneity in the way different stakeholders distinguish between assessment and appraisal. This creates ambiguity regarding the remit of the scientific assessment part in contrast to the more judgmental appraisal part. The new EU HTA Regulation (EU HTAR) has created an increased urgency to address this question since the regulation aims at harmonizing clinical technology assessment at the EU level, while technology appraisal remains in the remit of Member States [6]. Therefore, the field should seek clarity about the boundaries between assessment and appraisal.

## 2. How Are the Terms Assessment and Appraisal Used by Stakeholders?

There are no unique, generally accepted definitions for assessment and appraisal. Table 1 presents an overview of published terminology by major HTA stakeholders. In general, assessment is described as a predominantly technical and scientific exercise applying well-established methods and standards, guided by a desire to achieve maximum scientific objectivity. The various characterizations of appraisal are rather variable and often imprecise. The focus is on the more subjective interpretation of the evidence in a local healthcare context and on the judgment of added benefit. However, consistency on how assessment outcomes are translated into appraisals and how additional benefit is defined seems to be lacking. Some stakeholders call out explicit dimensions that should be considered such as clinical benefit or value for money, and a few appraisal definitions mention procedural aspects such as the use of stakeholder deliberation panels. Interestingly, none of the identified definitions mentions how to deal with uncertainty in the valuation of a technology.

The EUnetHTA definitions, where assessment refers to the “technical and scientific evaluation of the evidence” and appraisal refers to the “valuation of the assessment results that supports decision-making”, may serve as a starting point for more harmonized terminology [7]. On one hand, proposals by EUnetHTA can be seen as some sort of European HTA agency consensus. On the other hand, these succinct versions condense the essence of the various definitions that we found. Nevertheless, the field should seek more clarity on the distinction between the two terms and may need to be more explicit, particularly in the characterization of technology appraisal.

Technology appraisal includes—and often culminates in—the valuation of the totality of evidence by means of an ordinal categorization system. For example, the German Joint Federal Committee (Gemeinsamer Bundesausschuss, G-BA) rates the extent of added clinical benefit in six categories from “major” to “less benefit”, relative to the appropriate comparator therapy [8]. Similarly, the French National Authority for Health (Haute Autorité de Santé, HAS) qualifies the added clinical value of an intervention by assigning so-called Clinical Benefit Levels (ASMR, Amélioration de Service Médical Rendu), ranging from ASMR1 (major) to ASMR5 (no clinical improvement) [9]. Note that these appraisals go beyond a purely clinical judgment. A comparison of the HAS ratings with the outcomes of the American Society of Clinical Oncology Value Framework (ASCO-VF) and the European Society for Medical Oncology-Magnitude of Clinical Benefit Scale (ESMO-MCBS) revealed only weak correlation with these two scales (whilst the correlation between the ASCO-VF and the ESMO-MCBS was about twice as high, though still only moderate) [10]. However, within Europe, only a subset of countries have developed such categorization systems with explicit rules for the appraisal of new drugs [11,12,13]. This constitutes a major hurdle for the timely translation of evolving EU-level assessments into national appraisals and requires urgent action within the Member States.

**Table 1:** Definitions of the terms ‘assessment’ and ‘appraisal’ by key HTA stakeholders.

Source	Assessment	Appraisal
HTA Glossary.net [14]	A scientific process used to describe and analyse the properties of a health technology—its safety, efficacy, feasibility and indications for use, cost and cost-effectiveness, as well as social, economic and ethical consequences.	The process of assessing and interpreting scientific research results by systematically analysing their validity, clinical and statistical significance, and clinical relevance. [Definition of “Critical Appraisal”]
ISPOR HTA Council Working Group [15]	HTA […] may be viewed as informing evidence-based decision-making […]. The process of rigorous review and synthesis of scientific evidence focuses on assessing the relative benefits, harms, and costs of healthcare technologies using sound analytic judgments.	Evidence-based decision-making, in most cases, explicitly or implicitly incorporates other considerations (eg, affordability, ethical issues, feasibility, and acceptability) that may require mechanisms of contextualization of assessment results, such as deliberative processes, to support them.
EUnetHTA [7]	Technical and scientific assessment	Valuation of the assessment results that supports decision-making
EFPIA [16]	Factual relative effectiveness assessment	Translation of the factual evidence assessment into an added therapeutic value rating
EUPATI [17]	Synthesis and critical review of scientific evidence	Advice or recommendation considering the assessment in light of wider factors related to the local context
NICE Glossary [18]	A review of the evidence about how well health technologies work and how much value for money they present. The assessment report forms the basis of the evaluation committee’s discussions. The assessment report is written by an assessment group. Assessment reports are produced for treatments being assessed using the multiple technology appraisal process. [Definition of: “Assessment report”]	Formal assessment of the quality of research evidence and its relevance to the topic being considered. It is assessed according to predetermined criteria. [Definition of “Appraisal of evidence”]
Sandman and Heintz [19]	Action of evaluating relevant aspects of the technology to form a basis for decision	Implies some form of recommendation about the implementation of the technology, based on the assessment.
Angelis, Lange, and Kanavos [20]	Assessment of evidence conducted by technical groups	Appraisal of the assessed evidence from an expert committee that is producing reimbursement and coverage recommendation(s) for the final decision body, which can be either the payer, or the HTA agency itself. […] special considerations/social value judgements [are] applied in the appraisal phase
Wranik, Jakubczyk, and Drachal [21]	Review and quality rating of evidence that is guided by well-developed scholarly standards	Collective judgment by committee members about the clinical benefit and value for money of the therapy based on the considered evidence package
Fontrier, Visintin, and Kanavos [22]	Assessment refers to a process of collecting, reviewing and synthesising clinical and economic evidence to support funding decisions	Appraisal uses the same clinical and economic evidence but interprets it in the context of the healthcare system in question and takes into account factors that may be of relevance in that context
Patera and Wild [23]	Collection and synthesis of evidence; Method with focus on traceability/replicability of the results.	Contextualizing evidence and formulation of recommendations

## 3. How Does This Relate to the European HTA Regulation?

Bundling of competency for the clinical assessment of health technologies at the EU level is at the core of the EU HTAR. This process shares similarities with the centralized benefit–risk assessment performed by the European Medicines Agency, though with the important difference that health technology appraisal as well as subsequent reimbursement decision making fully remains in the remit of Member States. The EU HTAR aims at harmonizing the clinical assessment of health technologies at the EU level to reduce duplication and inconsistencies, remove inefficiencies, and foster innovation. During the development of the regulation, the European Parliament “called on the Commission to propose legislation on a European system for HTA […] to harmonise transparent HTA criteria in order to assess the added therapeutic value and relative effectiveness of health technologies […]” (EU HTAR preamble §9 [6]). This shows the ambition of the European Parliament to develop a European HTA system that provides meaningful guidance for subsequent therapeutic value categorization. The regulation also clarifies that Joint Clinical Assessment (JCA) reports “shall not contain any value judgement or conclusions on the overall clinical added value of the assessed health technology” (EU HTAR Article 9 [6]); in other words, technology appraisal stays fully in the competency of Member States.

On the path towards European HTA methodological standards, the Member State Coordination Group on Health Technology Assessment (HTA CG) develops guidelines for JCA building on the proposals from the EUnetHTA21 consortium [24,25]. On 25 March 2024, the HTA CG published a Methodological and a Practical Guideline on Quantitative Evidence Synthesis [26,27]. These guidelines (like the EUnetHTA21 proposals) put strong emphasis on the Member States’ competence to draw conclusions and make judgments. The HTA CG seems to translate Article 9 from the EU HTAR into an instruction to leave all judgments–of whatever kind–to the Member States. For example, the practical guideline states that “each Member State should be enabled to decide on the validity of direct or indirect treatment comparisons itself” [27]. This puts the objective of the EU HTAR to harmonize technology assessment at the EU level at risk. The scientific validity of the evidence should be judged at the EU level. Otherwise, the evidence base deemed scientifically valid for decision making may differ between Member States. This does not imply that the same evidence will be used for decision making in all countries and in the same way. But one should avoid situations where, for example, one country concludes that an indirect comparison presented by a developer is scientifically not valid and cannot be used, while another country bases major decisions on the same indirect comparison. Reimbursement decisions can and will differ between countries. But the evidence base deemed scientifically valid and therefore suitable as the basis for decision making should be established according to internationally accepted methods and best practices developed by the broader HTA community. This means that assessment involves scientific judgments about the validity and robustness of the evidence. As a result, JCA reports should adhere to commonly accepted rigors for methods and clear criteria for the reporting of scientific evidence that allow for the development and application of standards for an appraisal exercise; otherwise, they will not achieve the ambition of the EU HTAR. Furthermore, as decision making at a national level is multi-factorial, contextual factors on the disease and clinical practice, as well as the rationale for the request of a specific comparison, should be included at a European level to support national appraisal.

In addition, the specific local setup and organization of HTA and reimbursement decision making itself can influence the local judgment of scientific validity. This distorts the science/value dichotomy intended by separating assessment and appraisal. For evidence-based decision making to work properly, evidence assessment should be clearly separated from evidence appraisal, with different institutional bodies performing and leading each of the two stages, with an efficient interface so as not to cause delays [28].

Finally, for EU-level validity assessment to be meaningful, agreement on the cornerstones of the assessment as outlined in the decision scope formalized in the PICO (population, intervention, comparison, outcomes) scheme is paramount. The EUnetHTA21 “PICO pilots” revealed the challenges in achieving a workable pan-European decision scope that strikes the balance between European harmonization and local preferences [29,30,31]. Defining a set of EU-level PICOs that is actionable and useful for all parties will likely be more difficult than agreeing on suitable assessment methodology. For example, the list of comparators in an EU PICO should be reasonable and concise, based on clinical practice evidence, and giving priority to established medicines with robust clinical data and recommended in up-to-date European clinical guidelines [32].

## 4. Why Are Quantifying and Judging Uncertainty Instrumental?

Marketing authorization as well as reimbursement decisions for medical innovation must be made with imperfect information, leading to ethical issues and the question of how to deal with uncertainty [33]. For the regulatory context, Eichler et al. point out that entities such as the European Medicines Agency or the Food and Drug Administration have to judge how much risk is acceptable in the light of an expected benefit, as well as how much uncertainty around the expected benefit is acceptable at the time of marketing authorization [34]. Eichler et al. show that risk aversion by regulators comes with opportunity costs: an unwillingness to accept uncertainty will result in the suboptimal allocation of resources (for example, for clinical research) and, therefore, a loss in population health benefit. Therefore, these authors argue that “regulatory decisions should most closely reflect the preferences and degree of risk acceptable to patients (or their caregivers)” [34].

In contrast, HTA bodies typically inform resource allocation decisions for (potentially different types of) public money (be it tax-based or funded through mandatory health insurance, for example). In such setups, the full population bears the opportunity cost of funding a specific intervention. Therefore, the attitude towards uncertainty and risk may differ between HTA and regulatory agencies: HTA bodies should adopt in their decision making societal values and attitudes towards clinical benefit, unmet need, and willingness to accept risk [35]. In this process, the first step of technology assessment involves using HTA methodology to help reduce uncertainties as well as characterizing and quantifying the remaining uncertainties associated with the available evidence. The second step requires appraisal committees to rate the level, internal validity, and robustness of evidence from HTA reports based on established common criteria. Subsequently, technology appraisal provides additional context and local information, for example, on the uncertainty a Member State is willing to accept. This second part may include factors such as the burden and severity of the condition, local treatment alternatives, financial and budget considerations, and social, ethical, legal, and organizational factors [36,37,38].

Tools such as GRADE (Grading of Recommendations Assessment, Development and Evaluation) structure and standardize the evaluation and rating of the quality of clinical evidence [39,40]. The GRADE Evidence to Decision (EtD) framework extends this process to also capture decision making [41,42]. The first step in GRADE EtD is to clearly formulate the decision problem using the PICO scheme. Such frameworks could be further developed and tailored to the HTA and reimbursement decision space to establish best practices for technology appraisal [43,44]. Countries and decision makers may mainly differ in their willingness to accept risk, their evaluation of the unmet need for a given condition, their willingness (and ability) to pay for improvements in health, and in the importance given to other factors such as health equity impacts. However, countries should not per se differ in the rating of scientific dimensions covered by GRADE such as the internal validity of a study (though the applicability of evidence to a local context may differ), and the certainty of evidence. In the European context, appraisal is per se not in the scope of the EU HTAR. However, this should be an area for future voluntary collaboration and help to reduce Member State differences in benefit ratings that cannot be explained by the societal value dimensions listed above.

## 5. And What About Economic Evaluation?

People may argue that certain social value judgments are intrinsic to cost-effectiveness (CE) models, for example, in the choice of cost or effectiveness elements included in the model. Nevertheless, CE modeling also involves many scientific steps such as building an underlying disease model. In most cases, these scientific considerations hold at an above-country level and, therefore, could be centralized too. For example, health technology developers typically start by first building a global model, which is then adapted locally to reflect the decision problem (PICO) and perspective used by the decision maker. Country-specific epidemiology and cost data are applied too. This approach ensures that the underlying disease model and assumptions are implemented consistently across countries that use CE models in their decision making.

A recent comparison of economic evaluations of oncology drugs in Canada, the UK, and Australia showed that the corresponding HTA agencies reported largely the same basic elements of the submitted CE models and also identified similar methodological criticisms [45]. The guidelines for CE modeling are largely similar between these agencies, which corroborates the idea of a common scientific core also to economic modeling, which could be part of a joint collaborative assessment step [45]. CE models are per se out of the scope of the joint clinical assessments but EU HTAR explicitly foresees voluntary cooperation on areas such as the non-clinical domains of HTA (Article 23 [6]). Full economic evaluation as well as reimbursement decisions must then happen separately at the local level. Given the differences in healthcare pathways across Europe, the exact role of CE modeling in decision making also differs and implies a need for flexibility and adaptability of economic modeling at this last step.

Various other international networks seek to broaden collaboration even more and investigate cooperation in technology assessment, appraisal, and even pricing and reimbursement. For example, the CANZUK countries (Canada, Australia, UK, and New Zealand) set up a network for HTA collaboration with a focus on methodology development [46]; the Joint Nordic HTA-Bodies collaboration between the Danish, Finish, Norwegian, and Swedish HTA bodies aims at producing joint assessment reports that contain both clinical and economic assessments [47]; and the BeNeLuxA network envisions collaboration covering the full range from horizon scanning up to pricing and reimbursement [48]. However, such broader cooperation remains controversial and is beyond the scope of this work.

As we have been arguing so far, appraisal is quite different from assessment, and the harmonization of appraisals across jurisdictions is (much) more difficult. Nevertheless, more alignment in the scientific aspects of economic modeling (such as the underlying disease model) and guidance on the assessment of such models is, in principle, possible. In the long-term, this may lead to more harmonization (and, at some point, to a joint categorization system for added benefit decisions).

## 6. Conclusions: Embracing the Science vs. Value Dichotomy

Structuring reimbursement decision making into two stages, a first scientific step (the assessment), followed by a categorization of the overall added benefit in a local context (the appraisal), helps create transparency and consistency (Table 2). Scientific assessment should be guided by internationally accepted principles and methodological standards with (normative) objectives such as transparency, objectivity, and reproducibility in mind. Given the importance and impact of (potentially negative) reimbursement decisions, this also simplifies structured communication and, hopefully, increases their acceptability. Inevitably, decisions will be based on imperfect information, and the appraisal must therefore also address the relevance and management of the remaining uncertainty [35,49].

EU HTAR also builds on the assessment/appraisal dichotomy. However, we argue that further clarification of the terminology and a clear differentiation of the two concepts are needed. The successful implementation of the EU HTA Regulation needs further development of a common methodological basis reflecting current internationally accepted standards and best practices. The remit of EU HTA is scientific assessment. The HTA CG Subgroup on Methods is developing related guidance to evaluate the underlying evidence base. Consequentially, the application of those methods within the assessments and the related scientific judgments falls within EU HTA remit. In contrast, any ‘value judgments’ such as the translation of the scientific evidence in appraisal categories remain within the national remit. To meet those upcoming translational requirements, EU Member States urgently need to adopt transparent national appraisal procedures.

We emphasize that the explicit procedural steps and dimensions of the appraisal process can and should be standardized among Member States too. The resulting final ratings from this categorization process in terms of relevance and weightings given to the different dimensions of the appraisal process are context-driven and may vary among Member States. For the scientific scope on the EU level, the full evidence-based medicine triad should be involved to avoid misusing (arguably objective) assessment methods as means to impose singular preferences and implicit judgments [50]. Clinical expertise, patient preferences, and the totality of best available evidence and HTA methodology should all be combined to achieve EU-level scientific excellence in joint HTA. In the long term, European JCAs should become the primary scientific qualification of the evidence base suitable for localized contextualization and added value judgments by Member States.

## Figures and Tables

**Table 2:** Summary scheme of the regulatory, EU-level HTA, and local HTA evaluation.

	Regulatory	EU-Level HTA	Country-Level HTA
Group	European Medicines Agency (EMA), Committee for Medicinal Products for Human Use (CHMP)	Member State Coordination Group on Health Technology Assessment	Country-level HTA organization
Type of evaluation	Evaluation for marketing authorization	Joint Clinical Assessment (JCA)	National assessment (complementary clinical data), economic evaluation and non-clinical HTA domains (ethical, social, organizational, legal) National appraisal
Key outputs	European Public Assessment Report (EPAR)	JCA report	Local HTA (incl. appraisal) reports
Characteristics	Clinical efficacy/safety/quality Risk–Benefit	Scientific analysis of the relative effects and the degree of certainty Scientific judgments/Objective	Added value in local context Social value judgments Informs decisions (reimbursement and pricing)
